# Anemia and Red Blood Cell Transfusions, Cerebral Oxygenation, Brain Injury and Development, and Neurodevelopmental Outcome in Preterm Infants: A Systematic Review

**DOI:** 10.3389/fped.2021.644462

**Published:** 2021-02-26

**Authors:** Willemien S. Kalteren, Elise A. Verhagen, Jonathan P. Mintzer, Arend F. Bos, Elisabeth M. W. Kooi

**Affiliations:** ^1^Division of Neonatology, Department of Pediatrics, Beatrix Children's Hospital, University Medical Center Groningen, University of Groningen, Groningen, Netherlands; ^2^Department of Neonatology, Amsterdam University Medical Center, Vrije Universiteit Amsterdam, Amsterdam, Netherlands; ^3^Division of Newborn Medicine, Department of Pediatrics, Mountainside Medical Center, Montclair, NJ, United States

**Keywords:** anemia, prematurity, cerebral oxygenation, neuroimaging, neurodevelopmental outcome

## Abstract

**Background:** Anemia remains a common comorbidity of preterm infants in the neonatal intensive care unit (NICU). Left untreated, severe anemia may adversely affect organ function due to inadequate oxygen supply to meet oxygen requirements, resulting in hypoxic tissue injury, including cerebral tissue. To prevent hypoxic tissue injury, anemia is generally treated with packed red blood cell (RBC) transfusions. Previously published data raise concerns about the impact of anemia on cerebral oxygen delivery and, therefore, on neurodevelopmental outcome (NDO).

**Objective:** To provide a systematic overview of the impact of anemia and RBC transfusions during NICU admission on cerebral oxygenation, measured using near-infrared spectroscopy (NIRS), brain injury and development, and NDO in preterm infants.

**Data Sources:** PubMed, Embase, reference lists.

**Study Selection:** We conducted 3 different searches for English literature between 2000 and 2020; 1 for anemia, RBC transfusions, and cerebral oxygenation, 1 for anemia, RBC transfusions, and brain injury and development, and 1 for anemia, RBC transfusions, and NDO.

**Data Extraction:** Two authors independently screened sources and extracted data. Quality of case-control studies or cohort studies, and RCTs was assessed using either the Newcastle-Ottawa Quality Assessment Scale or the Van Tulder Scale, respectively.

**Results:** Anemia results in decreased oxygen-carrying capacity, worsening the burden of cerebral hypoxia in preterm infants. RBC transfusions increase cerebral oxygenation. Improved brain development may be supported by avoidance of cerebral hypoxia, although restrictive RBC transfusion strategies were associated with better long-term neurodevelopmental outcomes.

**Conclusions:** This review demonstrated that anemia and RBC transfusions were associated with cerebral oxygenation, brain injury and development and NDO in preterm infants. Individualized care regarding RBC transfusions during NICU admission, with attention to cerebral tissue oxygen saturation, seems reasonable and needs further investigation to improve both short-term effects and long-term neurodevelopment of preterm infants.

## Introduction

Anemia, described as low hemoglobin (Hb) or hematocrit (Ht) levels, is a common comorbidity in preterm infants in the neonatal intensive care unit (NICU) (1). The causes are multifactorial and include an immature hematopoietic system resulting in poor iron stores, decreased red blood cell (RBC) lifespan, low erythropoietin levels, and frequent blood sampling (2–4). Anemia is often poorly tolerated, resulting in tachycardia, apneic events, and poor feeding, and growth. Furthermore, it has been described that apparently stable anemic preterm infants increase their cardiac output up to 48 h after a transfusion. Though uncommon, this increases the risk of the development of left ventricular dysfunction (5).

When untreated, severe anemia may adversely affect organ function due to inadequate oxygen supply, possibly resulting in anemic tissue hypoxia and injury (6). Anemia may also result in alterations in cerebral oxygenation (7) and an increased risk for cerebral injury (8, 9). Existing data raise concerns about the impact of anemia on both short- and long-term neurodevelopmental outcome (NDO). The underlying mechanisms for neurodevelopmental sequelae are multifactorial and incompletely understood, but known causative factors include cerebral hypoxia, ischemia, oxidative injury, and fluctuations in cerebral perfusion (10–12).

Adequate neurologic development requires optimal oxygen supply to the central nervous system (13, 14). Anemia is usually treated with RBC transfusions to improve both short-term symptoms and long-term neurodevelopment. RBC transfusions increase red cell mass and oxygen-carrying capacity, although transfused adult RBCs have lower affinity for oxygen than fetal Hb, and thus lower the relative concentration of fetal Hb which may disrupt preterm homeostasis causing a potential decrease in cerebral blood flow (CBF) (15).

It has been estimated that more than 90% of extremely low-birth-weight infants receive one or more RBC transfusions during their NICU stay (3, 16). Transfusion thresholds remain controversial as RBC transfusions are associated with increased risk for ischemia-reperfusion damage or oxidative injury potentially resulting in transfusion-associated necrotizing enterocolitis, bronchopulmonary dysplasia and retinopathy of prematurity (1, 16). Several studies comparing high (liberal) and low (restrictive) Hb or Ht thresholds for RBC transfusion have been published (17–20), but controversies about when to transfuse anemic preterm infants still remain (21–23).

Near-infrared spectroscopy (NIRS) allows continuous, non-invasive monitoring of regional tissue oxygen saturation (rSO_2_) reflecting oxygen supply and metabolism (24, 25). The fractional tissue oxygen extraction (FTOE) reflects the balance between oxygen supply and consumption in the measured organ, taking the arterial oxygen saturation into account. It has been suggested that NIRS monitoring can provide relevant real-time data to assist in bedside decision-making regarding the hemodynamic status of an individual patient and to monitor the effect of therapeutic interventions such as RBC transfusions (26, 27).

This article provides a systematic review on the impact of anemia and RBC transfusions during NICU admission on neonatal cerebral oxygenation, measured using NIRS, and its association with brain injury and development and with neurodevelopmental outcomes in preterm-born children. In this systematic review, we present the literature published on this topic from the past 20 years.

## Methods

### Literature Search

This systematic review was performed according to the PRISMA guidelines for systematic reviews (28). To include all relevant original research articles for this review, we performed three separate PUBMED/EMBASE database searches independently by 2 authors (WSK and EMWK). Publications from January 1, 2000 to December 31, 2020 containing data on the impact of anemia and RBC transfusions on NIRS-based cerebral oxygenation, and/or brain injury and development, and/or NDO were selected. The complete search string of all three searches is available in the Supplementary Material.

Initial record titles were screened for relevance and abstracts of those records of potential relevance were reviewed. The third selection was based on the full-text of selected articles. Articles were included if they were written in English, contained original research in human subjects, focused on preterm neonates, and if at least part of the study group had anemia and/or received an RBC transfusion. Furthermore, cerebral oxygenation had to be assessed utilizing NIRS. We excluded articles that focused on fetal anemia or fetal transfusions. Articles focusing on exchange transfusions, erythropoietin and specific iron-deficiency anemia were also excluded. In addition to the database search, we reviewed the reference lists of the selected articles for additional relevant studies.

### Quality Assessment

The quality of all selected cohort and case-control studies was assessed using the Newcastle-Ottawa Quality Assessment Scale. This assessment scale consists of 3 parts: selection, comparability, and outcome. Ratings of these 3 factors generate a score, ranging from 0–9 points, with 9 points for the highest quality. In addition, the quality of selected randomized controlled trials (RCTs) was assessed using the Van Tulder Scale for randomized controlled trials. This scale consists of 11 items for which 1 point can be acquired per item. Therefore, the total score ranges from 0 to 11, with 11 representing highest quality. The Van Tulder Scale is a scale tool that has been recommended by the Cochrane Collaboration Back Review Group for the methodological assessment of RCTs (29).

## Results

Our first search for anemia, RBC transfusions, and cerebral oxygenation resulted in 433 articles. The second search for anemia, RBC transfusions, and brain injury and development resulted in 514 articles. Our third search for anemia, RBC transfusions and NDO produced 2,370 articles. After removing duplicates, a total of 2,645 articles remained. We excluded 2,550 articles based on titles alone. Reasons for exclusion were pre-clinical/non-human studies or studies focusing on fetal anemia or anemia resulting from iron-deficiency.

**Figure 1:**
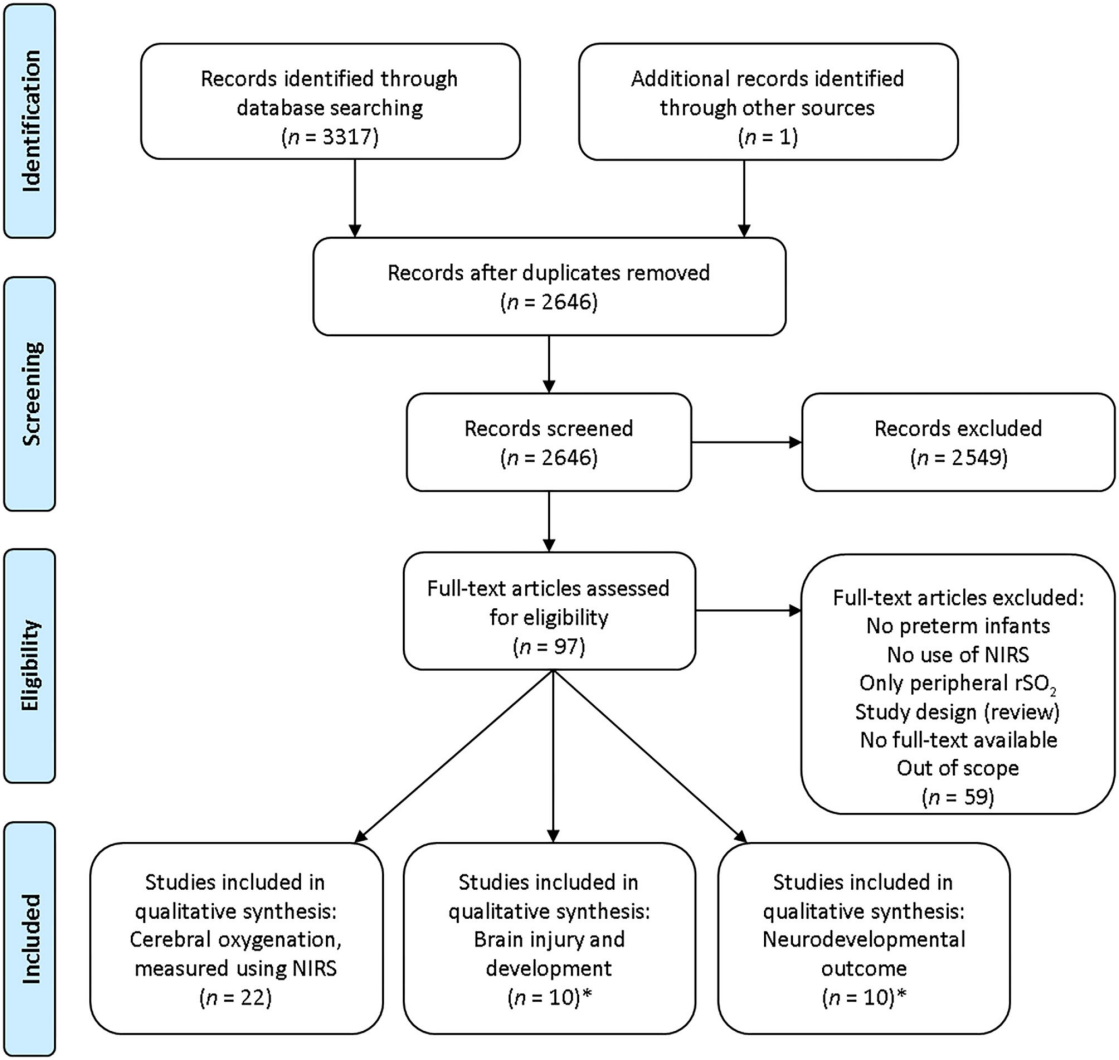
Flow diagram search strategy. NIRS, near-infrared spectroscopy; rSO_2_, regional tissue oxygen saturation.*Four articles were eligible for both outcome 2 and 3.

Abstracts or full-text articles were assessed within the remaining 96 articles. By analyzing the reference lists of the remaining articles, we included one additional article. Fifty-nine articles were additionally excluded due to the following: no data on cerebral oxygenation, not based on preterm infants, being a review article, or no full-text publication available. Four articles were eligible for both outcome two and three. Finally, 38 studies were included in our systematic review (Figure 1): 22 studies on cerebral oxygenation (7, 15, 30–49), 10 on brain injury and development (17–20, 50–55), and 10 on neurodevelopmental outcome (17, 18, 50, 53, 56–61). Characteristics of these articles are presented in Tables 1–**3**. Quality assessment scores are presented in Supplementary Tables 1–3.

**Table 1:** Results of selected studies on cerebral tissue oxygenation, measured by NIRS.

**References**	**Study design, No. infants**	**GA/BW**	**Study population**	**Outcome measure**	**RBC transfusion practice**	**Hb- / Ht-level**	**Cerebral oxygenation**
Goldstein et al. (30)	Cohort study, *n* = 31	<1,500 g and <35 wks	Anemic infants in need for RBC-tx	R_c_SO_2_; 1 h < RBC to 24 h > RBC	10–15 ml/kg in 3–5 h	Mean Ht increased from 31.4 to 37.4%	Anemia was associated with lower r_c_SO_2_; R_c_SO_2_ during and after RBC-tx did not differ by anemia status
Whitehead et al. (31)	Cohort study, *n* = 39	<30 wks	From 2nd wk PNA through 36 wks PMA	cFTOE; 8 h weekly	Volume NA; administered in 2 h	Median Hb was 9.9 g/dL; 69% had a measured Hb-level below 10 g/dL	Anemia was associated with critically increasing cFTOE, occurring at Hb-level of 9.6 g/dL
Jani et al. (32)	Cohort study, *n* = 40	<32 wks	Anemic infants in need for RBC-tx	R_c_SO_2_; 2 h < RBC to 4 h > RBC, and 24 h > RBC	15 ml/kg in 4 h	Mean Hb increased from 9.7 to 13.0 g/dL	Using liberal transfusion thresholds did only show a trend toward increasing r_c_SO_2_ after RBC-tx; Differences were more pronounced 24 h later
Aktas et al. (33)	Cohort study, *n* = 35	<33 wks	Anemic infants in need for RBC-tx	R_c_SO_2_; 24 h < RBC and 24 h > RBC	15–20 ml/kg in 3 h	Median Hb increased from 7.8 to 11.0 g/dL	R_c_SO_2_ was mostly maintained within normal limits during anemia and increased non-significantly after the RBC-tx
Jain et al. (34)	Cohort study, *n* = 30	<32 wks	Anemic infants in need for RBC-tx	R_c_SO_2_; 1 h < RBC and 1 h > RBC	15 ml/kg in 3 h	Mean pre-RBC-tx Hb was 9.8 g/dL	Mean r_c_SO_2_ increased after RBC-tx and was correlated with anemia severity and cardiac output-weighted oxygen delivery index
Saito-Benz et al. (35)	Cohort study, *n* = 24	<34 wks	Anemic infants in need for RBC-tx	R_c_SO_2_; 1 h < RBC, 24 h > RBC, and 5 d > RBC	15 ml/kg	Mean pre-RBC-tx Hb was 8.6 g/dL	RBC-tx led to an immediate increased r_c_SO_2_, but this change attenuated to baseline by 5 days
Kalteren et al. (36)	Case-control study, *n* = 8/16	<32 wks	Anemic infants in need for RBC-tx	R_c_SO_2_; 12 h > RBC	15 ml/kg in 3 h	Median Hb increased from 10.8 to 14.0 g/dL	R_c_SO_2_ and its variability remained stable during and after RBC-tx in infants that did not develop necrotizing enterocolitis
Mintzer et al. (37)	Cohort study, *n* = 27	<1,250 g	During first 10 days PNA	cFTOE; continuous	NA	Mean Ht was 39.7%	cFTOE was inversely correlated with Ht
Whitehead et al. (38)	Cohort study, *n* = 68	<30 wks	From 2nd wk PNA through 36 wks PMA	R_c_SO_2_; 8 h weekly	15 ml/kg	Median Hb was NA; 68% had a measured Hb-level below 10 g/dL	Increasing degree of anemia with progressive decrease in r_c_SO_2_; Critical Hb threshold for r_c_SO_2_ desaturation was 9.5 g/dL
Li et al. (39)	Case-Control study, *n* = 45/10	<32 wks	Anemic infants in need for RBC-tx; controls	R_c_SO_2_; 1.5 h < RBC to 2 h > RBC	Volume NA; administered in 3 h	Pre-RBC-tx Hb was below 14.4, 12.0, or 9.0 g/dL	Anemia reduces brain oxygen supply gradually to anemia severity; During and following RBC-tx r_c_SO_2_ peaked and remained stable
El-Dib et al. (40)	Cohort study, *n* = 72	<1,500 g and <34 wks	During 1st wk PNA and once after 1st wk PNA	cFTOE; weekly	NA	Mean Hb was 12.4 g/dL	Hb significantly affected cFTOE; cFTOE increased with reduced Hb
Banerjee et al. (41)	Cohort study, *n* = 59	<34 wks	Anemic infants in need for RBC-tx	R_c_SO_2_; 15 m < RBC to 15 m > RBC	15 ml/kg in 3 h	Mean Hb increased from 11.2 to 13.0 g/dL (1–7 days PNA), vs. 10.3 to 13.5 g/dL (8–28 days PNA), and vs. 9.1 to 12.2 g/dL (>28 days PNA)	Mean r_c_SO_2_ increased following RBC-tx in 3 different PNA groups, more pronounced after 28 days PNA
Andersen et al. (42)	Cohort study, *n* = 24	<29 wks	Anemic infants in need for RBC-tx on 1st day PNA	cFTOE; 30 m < RBC and 60 m > RBC	15 ml/kg in 3 h	Mean Hb increased from 11.5 to 12.6 g/dL (low pre-RBC-tx cFTOE), vs. 12.0 to 13.3 g/dL (high pre-RBC-tx cFTOE)	RBC-tx lowered cFTOE in infants with high pre-transfusion cFTOE
Mintzer et al. (43)	Case-Control study, *n* = 10/9	<1,250 g	Infants receiving “booster-” RBC-tx 1st wk PNA; controls	R_c_SO_2_ and cFTOE; continuous for 7 d	15 ml/kg in 3–4 h	Mean Ht was 35.2% in transfused infants vs. 43.5% in non-transfused infants	RBC-tx increased r_c_SO_2_ and reduced cFTOE irrespective of pre-transfusion Ht; No changes in non-transfused neonates
Sandal et al. (44)	Case-Control study, *n* = 23/16	<30 wks	Anemic infants in need for RBC-tx > 1st month PNA; controls	R_c_SO_2_; 10 h < RBC to 10 h > RBC	15 ml/kg in 2–4 h	Mean pre-RBC-tx Hb and Ht were 8.7 g/dL and 25% in transfused infants (with a significant increase after RBC-tx) vs. 12.3 g/dL and 37% in non-transfused infants	R_c_SO_2_ was lower in anemic infants than controls; RBC-tx improved r_c_SO_2_ independent of transfusion duration
Koyano et al. (45)	Cohort study, *n* = 19	<1,250 g	Anemic infants in need for RBC-tx > 48 h PNA	R_c_SO_2_; 6 h < RBC and 2–6 h>RBC	10–28 ml/kg	Median Hb increased from 9.3 to13.7 g/dL	R_c_SO_2_ increased by RBC-tx; greater CBF decrease in low pre-transfusion Hb infants
Seidel et al. (46)	Cohort study, *n* = 76	<32 wks	Anemic infants in need for RBC-tx	R_c_SO_2_; 4 h < RBC, during RBC, 4 h > RBC and 24 h > RBC	80 * weight in kg * (desired Ht-current Ht)/donor-Ht ml in 4 h	Mean Ht increased from 27.6 to 48.3% (low pre-RBC-tx r_c_SO_2_), vs. 27.3% to 47.7% (high pre-RBC-tx r_c_SO_2_)	R_c_SO_2_ increase until 24h after RBC-tx; Higher r_c_SO_2_ increase and less frequent desaturations after RBC-tx in infants with lower pre-transfusion r_c_SO_2_ values; No correlation between baseline r_c_SO_2_ and pre-RBC-tx Ht
Bailey et al. (47)	Cohort study, *n* = 30	<37 wks	Anemic infants in need for RBC-tx > 5 d PNA	R_c_SO_2_; 20 m < RBC to 20 m > RBC and 12 h > RBC	15 ml/kg in 4 h	Mean Hb and Ht increased from 9.3 g/dL and 27.6% to 12.4 g/dL and 36.5%	R_c_SO_2_ increased after RBC-tx and remained elevated 12 h after it began; No correlation was found between r_c_SO_2_ and Hb-levels
Dani et al. (48)	Cohort study, *n* = 15	<30 wks	Anemic infants in need for RBC-tx	R_c_SO_2_; 60 m < RBC to 60 m > RBC	Mean 28 ml/kg at 5 ml/kg/h	Mean Ht increased from 27.1 to 43.3%	RBC-tx followed by increased r_c_SO_2_, decreased cFTOE and reduced CBF velocity
Van Hoften et al. (7)	Cohort study, *n* = 33	<35 wks	Anemic infants in need for RBC-tx	R_c_SO_2_; 1 h < RBC, 1 h > RBC and 24 h > RBC	15 ml/kg in 3 h	Median Hb and Ht increased from 11.1 g/dL and 31% to 13.5 g/dL and 40%	Following RBC-tx r_c_SO_2_ increased and cFTOE decreased quickly; R_c_SO_2_ might be at risk when Hb <9.7 g/dL
Dani et al. (15)	Cohort study, *n* = 14	<34 wks	Anemic infants in need for RBC-tx 7 d PNA to <1st month PNA	R_c_SO_2_; 30 m < RBC to 30 m > RBC	25 ml/kg at 5 ml/kg/h	Mean Hb and Ht increased from 9.1 g/dL and 28% to 14.6 g/dL and 45%	RBC-tx improves cerebral oxygen supply and decreases cerebral blood volume (increase cerebrovascular resistance)
Wardle et al. (49)	Case-Control study, *n* = 46/43	<32 wks	Anemic infants in need for RBC-tx; stable controls	cFTOE; 10 m once and 10 m 12–24 h > RBC	20 ml/kg	Median Hb increased from 12.3 to 15.2 g/dL; Hb-level in controls was 14.0 g/dL	cFTOE was similar between anemic infants and controls; After RBC-tx cFTOE decreased in transfused infants; cFTOE was inversely correlated with Hb

*NIRS, near-infrared spectroscopy; GA, gestational age; BW, birth weight; RBC-tx, red blood cell transfusion; NA, not applicable; r_c_SO_2_, cerebral regional tissue oxygen saturation; cFTOE, cerebral fractional tissue oxygen extraction; PNA, postnatal age; PMA, postmenstrual age; Hb, hemoglobin; Ht, hematocrit; CBF, cerebral blood flow; SaO_2_, arterial oxygen saturation; d, day; h, hour; m, minutes*.

### Anemia, RBC Transfusions, and Cerebral Oxygenation

The effect of anemia and/or RBC transfusions on cerebral oxygenation was described in 22 articles (Table 1), representing a total of 854 preterm infants. These studies were observational case-control studies or cohort studies that compared cerebral oxygenation in preterm infants either before and after RBC transfusion or at subsequent times during NICU admission.

Five studies described anemia of prematurity and cerebral oxygenation. In general, during the first weeks after birth an increasing degree of anemia with progressive decrease in cerebral rSO_2_ (r_c_SO_2_) or increase in cerebral FTOE (cFTOE) was reported (31, 38, 40, 41). Mintzer et al. found no changes in cerebral oxygen saturation and extraction in 9 non-transfused neonates during the first week after birth (43). In a further report, they reported Hb to be inversely correlated with cFTOE, with increasing cFTOE hypothesized as a potential early marker of nascent anemia during the first 10 days after birth (37). Similar correlations between Hb and cerebral rSO_2_ or cerebral FTOE were described in 5 other articles (38–40, 44, 49). Conversely, Seidel et al. (46) and Bailey et al. (47) found no correlation between r_c_SO_2_ and Hb-levels.

In preterm infants receiving RBC transfusions according to local protocols, anemia was associated with lower r_c_SO_2_ in most cases (30, 39, 40, 44), but Wardle et al. found similar cFTOE between anemic infants and controls (49). In the latter study, however, many babies were transfused based on physician discretion, rather than on the cFTOE cut-off levels mentioned in their study protocol. Whitehead et al. reported a critical Hb threshold of 9.5 g/dL before cerebral oxygen saturation declined (31, 38). Similar results were demonstrated by Van Hoften et al. who described diminished cerebral oxygen saturation and increased cFTOE with a Hb-level below 9.7 g/dL (7).

The majority (83%) of the 18 studies that reported on cerebral oxygenation during and after RBC transfusion found r_c_SO_2_ to be higher during and after RBC transfusion compared to pre-transfusion levels in anemic preterm infants (7, 15, 30, 34, 35, 39, 41–49). Non-significant changes in cerebral oxygen saturation during and after RBC transfusions were observed in 3 studies (32, 33, 36).

The effect of RBC transfusion on cerebral oxygenation parameters was mostly short-lasting. Increased r_c_SO_2_ remained elevated until 12 or 24-h following transfusion in several studies (7, 32, 46, 47). Twenty-four hours following RBC transfusion, an even greater difference was measured compared with pre-transfusion cerebral oxygenation (7, 32), especially in infants with the lowest pre-transfusion Hb (7). Saito-Benz et al. described an immediate increase in r_c_SO_2_, followed by an attenuated r_c_SO_2_ back to pre-transfusion levels, during the 5 days after the RBC transfusion (35).

In eight studies, the effect of pre-transfusion anemia severity on cerebral oxygenation was taken into account when assessing r_c_SO_2_ and cFTOE after RBC transfusion (7, 30, 34, 39, 42, 43, 45, 46). Goldstein et al. (30) and Mintzer et al. (43) found an increased r_c_SO_2_ and decreased cFTOE irrespective of the pre-transfusion Hb or Ht. All others described a correlation with anemia severity. In particular, Van Hoften et al. reported that infants with a lower Hb-level before RBC transfusion demonstrated a more pronounced effect on cerebral oxygenation parameters (7). Andersen et al. only observed lowered cFTOE following RBC transfusion in infants with higher pre-transfusion cFTOE (42), and Seidel et al. described a more pronounced r_c_SO_2_ increase following RBC transfusion when infants had lower pre-transfusion r_c_SO_2_ values (46).

### Anemia, RBC Transfusions, and Brain Injury and Development

The main findings regarding the effects of neonatal anemia and RBC transfusions on brain injury and development were reported in 10 studies, most typically consisting of preterm infants being followed-up after participation in liberal vs. restrictive RBC transfusion threshold randomized trials. Table 2 provides an overview of these studies. Brain injury during NICU admission was described in 6 studies (*n* = 3,602 infants). In four other studies, brain development was described either at school age (*n* = 95 children) or at 34–37 weeks postmenstrual age (PMA) (*n* = 21 infants).

**Table 2:** Results of selected studies on brain injury and development.

**References**	**Study design, No. infants**	**GA/BW**	**Study population**	**Outcome measure**	**RBC transfusion practice**	**Hb-/Ht-level**	**Brain injury and development**
Kirpalani et al. (17)	RCT, *n* = 1,824	<1,000 g and >22 to <29 wks	Preterm infants in liberal and restrictive RBC-tx group	Brain ultrasound	15 ml/kg	Pre-transfusion mean Hb differed between groups by 1.9 g/dL	No difference in percentage of infants with moderate or severe IVH, or PVL between infants randomized to liberal and restrictive transfusion thresholds
Fontana et al. (50)	Case-Control study, *n* = 178/182	<1,500 g and ≤ 32 wks	Transfused and non-transfused preterm infants	Brain ultrasound	10–15 ml/kg in 4 h	NA	Transfused infants showed a higher incidence of severe IVH and PVL
Franz et al. (18)	RCT, *n* = 1,013	>400 to <999 g and <30 wks	Preterm infants in liberal and restrictive RBC-tx group	Brain ultrasound	20 ml/kg	Mean Ht during 1st week was 39.5% (restrictive group) vs. 41.9% (liberal group); During 2nd week this was 36.2 vs. 39.5%	No difference in percentage of infants with moderate or severe IVH, or PVL between infants assigned to liberal and restrictive transfusion thresholds
Benavides et al. (51)	Follow-up study, *n* = 25	>500 to <1,300 g	Female liberal and restrictive transfusion threshold infants at school age	Brain MRI	15 ml/kg in 5 h	Mean Ht was 35.7% (restrictive group) vs. 44.3% (liberal group)	Liberal RBC-tx practice was associated with deficit of WM brain structure, with decreased temporal lobe and caudate structure
Morris et al. (52)	Cohort study, *n* = 21	<1,500 g	Anemic preterm infants; 34–37 wks PMA	Brain MRI	NA	Mean Ht was 31.3%	Higher CBF in infants with lowest Ht; Elevated oxygen extraction was associated with worsening anemia
McCoy et al. (53)	Follow-up study, *n* = 26	>500 to <1,300 g	Liberal transfusion threshold infants at school age	Brain MRI	15 ml/kg in 5 h	NA	Possible adverse effect of high RBC-tx thresholds in which females had decreased temporal lobe WM, related to poor verbal fluency
Nopoulos et al. (54)	Case-Control follow-up study, *n* = 44/40	>500 to <1,300 g	Liberal and restrictive transfusion threshold infants at school age; healthy term controls at school age	Brain MRI	15 ml/kg in 5 h	Mean Ht was 36.5% (restrictive group) vs. 44.8% (liberal group)	Liberal RBC-tx group had greatest brain structure abnormalities with decrements in ICV; Cerebral WM was more substantially reduced in liberal group; Liberal group girls had most abnormalities; Cerebral WM volume was inversely correlated with Ht-level
Chen et al. (55)	RCT, *n* = 36	<1,500 g	Preterm infants in liberal and restrictive RBC-tx group	Brain ultrasound	10 ml/kg	Mean Hb and Ht on day 30 after birth were similar between groups; 10.4 g/dL and 29.9%	No differences in percentage of infants with moderate or severe IVH
Kirpalani et al. (19)	RCT, *n* = 451	<1,000 g and <31 wks	Preterm infants in liberal and restrictive RBC-tx group	Brain ultrasound	15 ml/kg	Mean Hb during 1st week was 14.3 g/dL (restrictive group) vs. 14.9 g/dL (liberal group); During 2nd week this was 11.9 vs. 13.1 g/dL	Brain injury slightly favored the low threshold group non-significantly
Bell et al. (20)	RCT, *n* = 100	>500 to <1,300 g	Preterm infants in liberal and restrictive RBC-tx group	Brain ultrasound	15 ml/kg in 5 h	Mean Hb and Ht were 8.3 g/dL and 26% (restrictive group) vs. 11.0 g/dL and 32% (liberal group)	Restrictive RBC-tx group included more infants with IVH gr 4, and more infants suffering severe adverse brain events (IPL, PVL)

*GA, gestational age; BW, birth weight; RBC-tx, red blood cell transfusion; NA, not applicable; PMA, postmenstrual age; Ht, hematocrit; CBF, cerebral blood flow; WM, white matter; ICV, intracranial volume; RCT, randomized controlled trial; IVH, intraventricular hemorrhage; IPL, intraparenchymal brain hemorrhage; PVL, periventricular leukomalacia*.

Brain injury during NICU admission was assessed using brain ultrasound (17–20, 50, 55). Both Kirpalani et al. (17), Franz et al. (18), and Chen et al. (55) showed no differences in percentage of infants with moderate IVH, severe IVH, or PVL between infants assigned to liberal vs. restrictive RBC transfusion thresholds. Non-significantly less abnormalities were shown on brain ultrasound in the low threshold group (19). Interestingly, more infants with severe IVH and PVL were reported in the group of infants that received less RBC transfusions during the IOWA randomized controlled trial (20). A retrospective study observed a higher incidence of severe brain injury in transfused preterm infants vs. non-transfused infants (50).

Concerning brain development, regional brain measures assessed on brain MRI were mostly smallest in female study participants, and were inversely related to average Ht-level: those children with the highest neonatal average Ht-level were the ones with the lowest volumes of white matter and thalamic volume at 12 years (51, 53, 54). Liberal RBC transfusion practices were associated with reduced cerebral white matter at school age, especially within the temporal lobe and subcortical nuclei (51, 53, 54).

Brain MRI at near-term age (PMA range 34.0–36.9 weeks) showed increased fractional oxygen extraction in brain tissue in infants with lower Ht-levels, suggesting ongoing hemodynamic compensation for anemia (52).

### Anemia, RBC Transfusions, and Neurodevelopmental Outcome

Ten studies (both RCTs and observational) described a relationship between anemia and RBC transfusions during NICU admission and NDO (Table 3).

**Table 3:** Results of selected studies on neurodevelopmental outcome.

**References**	**Study design, No. infants**	**GA/BW**	**Study population**	**Outcome measure**	**RBC transfusion practice**	**Hb-/Ht-level**	**Neurodevelopmental outcome**
Kirpalani et al. (17)	RCT, *n* = 1,692	<1,000 g and >22 to <29 wks	Preterm infants in liberal and restrictive RBC-tx group; FU at 22–26 m PT	Bayley-III	15 ml/kg	Pre-transfusion mean Hb differed between groups by 1.9 g/dL	No difference in death or disability at 22–26 m PT between liberal and restrictive threshold groups; Liberal RBC-tx strategy did not improve survival without neurodevelopmental impairment
Fontana et al. (50)	Case-Control study, *n* = 178/182	<1,500 g and ≤ 32 wks	Transfused and non-transfused preterm infants; FU both at 2 y and 5 y PT	GMDS	10–15 ml/kg in 4 h	NA	RBC-tx are negatively associated with NDO with a cumulative effect; RBC-tx within 28 days is associated with greater reduction in NDO scores; Impact on NDO persists at 5 y of age
Franz et al. (18)	RCT, *n* = 843	>400 to <999 g and <30 wks	Preterm infants in liberal and restrictive RBC-tx group; FU at 24 m PT	Bayley-II or Bayley-III	20 ml/kg	Mean Ht during 1st week was 39.5% (restrictive group) vs. 41.9% (liberal group); During 2nd week this was 36.2 vs. 39.5%	No difference in death or disability at 24 m PT between liberal and restrictive threshold groups; Liberal RBC-tx strategy did not reduce likelihood of death or disability
Wang et al. (56)	Cohort study, *n* = 98	<1,000 g	Preterm infants; FU at 2 y PT	Bayley-II	10–15 ml/kg in 2–3 h	Mean initial Hb was 15.0 g/dL	Number of RBC-tx was negatively correlated with survival; Early RBC-tx (<7 d) was associated with higher Bayley scores
Velikos et al. (57)	Cohort study, *n* = 120	≤32 wks	Preterm infants; FU at 1 y PT	Bayley-III	NA	NA	Adjusted for other risk factors, number of RBC-tx was negatively correlated with Bayley scores
McCoy et al. (53)	Follow-up study, *n* = 26	>500 to <1,300 g	Liberal transfusion threshold infants; FU at school age	WISC-IV	15 ml/kg in 5 h	NA	Non-significant lower performances by females on all measures; Lower WM volume was associated with less verbal fluency
McCoy et al. (58)	Follow-up study, *n* = 56	>500 to <1,300 g	Preterm infants in liberal and restrictive RBC-tx group; FU at school age	WISC-IV	15 ml/kg in 5 h	Mean Ht was 36.7% (restrictive group) vs. 44.5% (liberal group)	Poorer cognitive outcomes on all intelligence assessments and neuropsychological tests in liberal RBC-tx group
von Lindern et al. (59)	Cohort study, *n* = 67	<28 wks	Preterm infants in different RBC-tx volume groups; FU at 24 m PT	Bayley-II	15 vs. 20 ml/kg	NA	No relation between NDO at 24 m PT and transfusion volume during NICU admission
Whyte et al. (60)	Follow-up study, *n* = 421	<1,000 g and <31 wks	Preterm infants in liberal and restrictive RBC-tx group; FU at 18–21 m PT	Bayley-II	15 ml/kg	NA	No difference in composite outcome of death or NDI; Cognitive delay defined as <85 favored the liberal threshold group
Gabrielson et al. (61)	Follow-up study, *n* = 43	<29 wks	Preterm infants; FU at school age	WISC-III	NA	NA	Low performance IQ was associated with high number of RBC-tx; Infants with higher RBC-tx rates had similar or higher verbal IQ scores than performance IQ

*GA, gestational age; BW, birth weight; FU, follow-up; PT, post-term; NA, not applicable; Bayley-II, Bayley Scales of Infant and Toddler Development, 2nd edition; Bayley-III, Bayley Scales of Infant and Toddler Development, 3rd edition; GMDS, Griffiths Mental Development Scales; RBC-tx, red blood cell transfusion; WISC-IV, Wechsler Intelligence Scale for Children, 4th edition; WM, white matter; RCT, randomized controlled trial; m, months; y, years; NDI, neurodevelopmental impairment; WISC-III, Wechsler Intelligence Scale for Children, 3rd edition*.

Focusing only on the RCTs, there were 4 clinical trials comparing liberal and restrictive RBC transfusion strategies in which a total of 2919 children participated. The first by Kirpalani et al. was the TOP trial in which they found no differences in NDO at 22–26 months corrected age between preterm infants randomized to either liberal or restrictive transfusion thresholds (17). Another recently published RCT was the ETTNO trial by Franz et al. in which NDO was determined at 24 months corrected age in ELBW neonates (18). No significant differences in NDO were observed between the liberal and restrictive transfusion groups. Whyte et al. assessed NDO at 18–21 months corrected age in ELBW infants who originally participated in the PINT study (60). At follow-up, they observed a lower cognitive outcome in preterm-born children treated with a restrictive transfusion strategy. McCoy et al. reported NDO at 8 to 15 years of age in preterm-born children (58). Children transfused under the liberal strategy performed less on associative verbal fluency, visual memory and reading compared to children treated under the restrictive transfusion strategy. Furthermore, in a follow-up analysis, they found lower verbal fluency in preterm born female children at an average age of 13 years compared to preterm born male children (53).

Three observational studies demonstrated that the number of RBC transfusions was correlated with lower NDO scores at both 2 and 5 years corrected age (50), with lower cognitive, language and motor scores at 12 months adjusted age (57), and with a lower performance IQ than verbal IQ at 8–11 years (61). In the fourth observational study by Wang et al. they observed a higher mental developmental index score at 18 and 24 months corrected age in 62 ELBW infants who received RBC transfusions within 7 days after birth (56). There was one study reporting a lack of effect of transfusion volume on NDO at 24 months' corrected age (59).

## Discussion

In this systematic review, we aimed to increase understanding of the impact of anemia and RBC transfusions on the developing brain of the preterm infant. This systematic review demonstrated that anemia of varying severity may reduce oxygen supply to the brain of preterm infants. RBC transfusions, on the other hand, improve oxygen supply to the brain. Infants with more severe anemia demonstrated a more pronounced short-term effect of an RBC transfusion, which is likely important for long-term outcomes by avoiding anemic hypoxic injury. Severe anemia during NICU admission seems to be associated with disturbances of brain development, even though findings on long-term outcome suggest potential neuroprotective benefits from a restrictive RBC transfusion threshold.

Cerebral oxygenation continues to demonstrate promise for predicting outcome in preterm infants (11, 62), as this measure reflects the integration of multiple parameters including oxygen delivery and oxygen demand and consumption (63).

### Anemia and Cerebral Oxygenation

In general, decreasing Hb-level correlated with either decreasing r_c_SO_2_ or increasing cFTOE (30, 37–40, 44, 49) A few studies described a critical Hb-threshold around 9.5 g/dL before cerebral oxygen saturation and extraction undergo noticeable changes (7, 31, 38). Furthermore, increased PNA was associated with lower Hb-levels and a progressive decrease in r_c_SO_2_ or increase in cFTOE (31, 38, 40, 41). In other studies, varying PMA might have prevented demonstration of a correlation between Hb-level and cerebral oxygenation (46, 47). Additionally, the duration of measuring cerebral oxygenation seems to be important. Wardle et al. did not find a difference in cFTOE between anemic infants and controls (49). However, cFTOE was measured for only 10 min in this study, as compared with measurements taken over hours by subsequent researchers.

### RBC Transfusions and Cerebral Oxygenation

As pre-transfusion baseline cerebral oxygen saturation decreases with increasing chronological age, it is likely that CBF and oximetry responses to RBC transfusion are dependent on chronological age in preterm infants. As expected, cerebral oxygen saturation and extraction in most cases were significantly affected by RBC transfusion. R_c_SO_2_ was higher during and up to 24 h after the RBC transfusion when compared to r_c_SO_2_ pre-transfusion levels (7, 15, 30, 34, 35, 39, 41–49). Cerebral oxygen saturation, however, attenuated to pre-transfusion values during subsequent days, questioning the clinical relevance of the briefly improved cerebral oxygenation. A possible explanation is the RBC transfusion leading to an increased preload, cardiac output and CBF. Over subsequent days, this enhanced CBF response may diminish with oxygenation parameters returning to pre-transfusion values (35). Another explanation is the increased fraction of adult Hb in comparison with before RBC transfusion, thus reducing the fraction of fetal Hb with a shift in dissociation curve (15). Possible explanations for not finding a significant difference in cerebral oxygenation during and after RBC transfusion in several reports, may relate to liberal transfusion thresholds (32), missing r_c_SO_2_ data before RBC transfusions (36), or adequate cerebral autoregulation providing a constant CBF (33).

The effect of the RBC transfusion on cerebral oxygenation was more pronounced in infants with lower pre-transfusion Hb- or Ht-levels (7, 34, 39, 42, 45, 46). Increased oxygen extraction under baseline conditions leaves little reserve to meet the demands of brain tissue during oxygen desaturations. An explanation for not finding differences between pre-transfusion anemia severity might be the fact that peripheral tissues demonstrate a more robust response than the brain, possibly as a result of the neuroprotective maintenance of cerebral oxygen delivery (37, 48). This regulation of oxygen-carrying capacity to the brain might explain the findings of increased r_c_SO_2_ and decreased cFTOE after RBC transfusion irrespective of pre-transfusion anemia severity (30). Another possibility for these findings may be related to the effects of other RBC transfusion strategies, i.e., “booster” transfusions (43).

### Anemia, RBC Transfusions, and Brain Injury

Concerning brain injury, anemia has previously been associated with a significant increase in CBF (64), which has been posited as a risk factor for developing IVH (8). Conversely, if this compensatory mechanism fails, there could be an increased risk for hypoxic brain injury. The association between RBC transfusion strategy and brain injury during NICU admission is still under debate. Most studies observed no difference in presence of brain injury between RBC transfusion strategies (17–19, 55). Conversely, Bell et al. (20) reported more infants with severe IVH and PVL following restrictive RBC transfusion thresholds, possibly because of rather low Hb- and Ht-levels in their restrictive RBC transfusion threshold infants compared to mean Hb- and Ht-levels in other study participants (17–19).

### Anemia, RBC Transfusions, and Brain Development

Regarding brain development, this systematic review demonstrated more available evidence for brain structure abnormalities at school age among neonates transfused under liberal transfusion thresholds (51, 53, 54) Children with highest average Ht-levels had lowest brain volumes at 12-years of age, supporting the notion that the abnormalities are indeed related to Ht-level (and thus to transfusion status) (51, 53, 54). Of note, all three follow-up studies describing brain MRI at school age included a sample of children that were initially enrolled in the same randomized controlled trial (20).

### Anemia, RBC Transfusions, and Neurodevelopmental Outcome

Similarly, available evidence supports a restrictive RBC transfusion strategy, showing a favorable NDO at school age among preterm infants randomized to lower RBC transfusion thresholds during NICU admission (17, 18, 53, 57, 58, 61). Apart from one study (60), this also holds true for NDO at 2 years corrected age.

There seems to be a discrepancy between short-term outcomes, NDO at 2 years of age, and long-term NDO at school age. A restrictive RBC transfusion strategy was associated with poorer short-term outcomes and with poorer NDO at 2 years' corrected age (20, 60), while longer-term outcomes may be adversely affected by liberal RBC transfusion strategies (53, 54, 58). In light of the beneficial effect of a more restrictive strategy, the liberal transfusion group expectantly demonstrated the greatest abnormality in brain structure with significant decrements in intracranial volume (53, 54). However, data on the relationship between brain structure in school-aged children originally assigned to the restrictive transfusion strategy are lacking. These authors speculate that a lack of endogenous erythropoietin in the liberal group may be associated with worse outcome. Endogenous erythropoietin is essential for the production of erythrocytes. Several studies have reported substantial neuroprotective properties of erythropoietin, functioning in the brain as both an important growth factor and a neuroprotective agent (65–68). RBC transfusions during NICU admission may result in less endogenous erythropoietin production. This suppression of erythropoietin may translate into “loss” of a growth factor known to promote brain growth and recovery from brain injury (66).

The results of this review suggest that a restrictive transfusion strategy is associated with better gain in Hb-level, oxygen delivery, and cerebral oxygen saturation following RBC transfusion. The preterm brain, however, is particularly vulnerable to hypoxic injury (69). Cerebral oxygenation may be at risk when Hb-levels decrease below 9.5 g/dL (7, 31, 38). Existing reference data on r_c_SO_2_ suggest reference values between 65 and 75% using an INVOS monitor in combination with neonatal sensors during the first week after birth (70–73). Furthermore, Verhagen et al. showed cerebral oxygenation between 72 and 83% to be associated with a favorable NDO (11). More recently, Alderliesten et al. also observed low cerebral oxygenation to be associated with poorer cognitive outcome, suggesting a threshold of approximately 65% using neonatal sensors (74). An increasing cFTOE may also indicate an early pathophysiological response to anemia (37, 52) and may serve as a potential biomarker for cerebral injury and long-term NDO in premature infants. Identification of the vulnerable subgroup of preterm infants with low cerebral oxygen saturation may be clinically important to administer RBC transfusions in a timely manner leading to better clinical outcomes. We confirm previous implications that RBC transfusions improve tissue oxygenation and that tissue oxygenation itself may play an important role in identifying the trigger for RBC transfusion (7, 15, 34, 44, 46, 47). Suboptimal precision of current NIRS measurements, however, preclude us from determining absolute thresholds (75).

This systematic review has several limitations. First, many included studies were observational in nature. These are associated with a risk of bias of either under- or overestimating outcome measures. Furthermore, inclusion of mainly observational studies makes it difficult to draw definite conclusions. Second, unless studied prospectively, infants who were assigned in both observational studies and RCTs investigating RBC transfusion strategies form a biased group. Almost all studies, however, were of reasonable to good quality according to the quality assessments. Finally, preterm infants requiring RBC transfusions were younger, smaller, sicker, and had more frequent inotropic treatments. Therefore, they already have a higher risk for morbidity and adverse NDO. A relatively large number of infants who had otherwise similar neonatal clinical conditions, however, were enrolled in all included publications.

## Conclusion

This systematic review suggests that anemia and RBC transfusions during NICU admission contributed significantly to brain development and NDO in preterm infants, possibly by its association with cerebral oxygenation. An individualized approach regarding RBC transfusion strategy using NIRS-based cerebral tissue oxygen saturation assessments in order to support brain growth and development and to prevent neurodevelopmental delay in anemic preterm infants seems reasonable. When combining the results of the aims for this review, one might suggest that when cerebral oxygen saturation drops below the levels associated with poorer NDO, i.e., below 65 or 70%, this insinuates the need for further evaluation to determine whether anemia is present. If Hb-level is low, this would warrant considering an RBC transfusion. Whether using a lower threshold of cerebral oxygen saturation to trigger RBC transfusion needs further prospective investigation.

## Data Availability Statement

The original contributions generated for the study are included in the article/Supplementary Material, further inquiries can be directed to the corresponding author/s.

## Author Contributions

WK conceptualized and designed the study, developed the search strategy, screened databases for eligible studies, assessed full-text articles for eligibility, conducted the quality assessment, drafted the initial manuscript, and revised the manuscript after feedback from coauthors. EV conceptualized and designed the study, drafted part of the initial manuscript, and critically reviewed and revised the manuscript. JM and AB conceptualized and designed the study, and critically reviewed and revised the manuscript. EK conceptualized and designed the study, provided support with the search strategy, screened databases for eligible studies, and critically reviewed and revised the manuscript. All authors approved the final manuscript as submitted.

## Conflict of Interest

The authors declare that the research was conducted in the absence of any commercial or financial relationships that could be construed as a potential conflict of interest.
