# Supplementary material for: Anemia and Red Blood Cell Transfusions, Cerebral Oxygenation, Brain Injury and Development, and Neurodevelopmental Outcome in Preterm Infants: A Systematic Review
Source: Front Pediatr. 2021 Feb 26;9:644462. doi: 10.3389/fped.2021.644462 (PMC7952449; doi:10.3389/fped.2021.644462)
Supplement: Supplementary file 1 [file Data_Sheet_1.docx]

Supplementary Material

*Search strategy*

For topic #1 we used the following search terms: ("Anemia"[Mesh] OR anemi*[tiab] OR anaemi*[tiab] OR "Blood Transfusion"[Mesh] OR blood transfus*[tiab] OR erythrocyte transfus*[tiab] OR red cell transfus*[tiab] OR red blood cell transfus*[tiab]) **AND** ("Infant, Newborn"[Mesh] OR prematur*[tiab] OR preterm[tiab] OR neonat*[tiab] OR newborn*[tiab] OR infant*[tiab] OR elbw[tiab] OR vlbw[tiab]) **AND** ("Oxygen Consumption"[Mesh] OR “Spectroscopy, Near-Infrared"[Mesh] OR near-infrared spectro*[tiab] OR NIRS[tiab] OR NIR spectro*[tiab] OR cerebral oxygen saturation[tiab] OR tissue oxygen*[tiab]).

For topic #2 we used the following search terms: ("Anemia"[Mesh] OR anemi*[tiab] OR anaemi*[tiab] OR "Blood Transfusion"[Mesh] OR blood transfus*[tiab] OR erythrocyte transfus*[tiab] OR red cell transfus*[tiab] OR red blood cell transfus*[tiab]) **AND** ("Infant, Newborn"[Mesh] OR prematur*[tiab] OR preterm[tiab] OR neonat*[tiab] OR newborn*[tiab] OR infant*[tiab] OR elbw[tiab] OR vlbw[tiab]) **AND** (“Head”[Mesh] OR “Cerebrovascular Circulation”[Mesh] OR “Brain/blood supply”[Mesh] OR “Brain/embryology”[Mesh] OR “Brain/abnormalities”[Mesh] OR “Brain/pathology”[Mesh] OR “Brain/physiopathology”[Mesh] OR “Cerebral Arteries”[Mesh] OR hypoxi*[tiab] OR ischemi*[tiab] OR brain*[tiab] OR cerebr*[tiab] OR transcranial[tiab]) **AND** (“Magnetic Resonance Imaging”[Mesh] OR “Imaging, Three-Dimensional”[Mesh] OR MRI[tiab] OR magnetic resonance[tiab] OR MR imaging[tiab] OR MR tomograph*[tiab] OR 3d imag*[tiab] OR three-dimensional imag*[tiab] OR imaging[tiab] OR amplitude integrated electroencephalo*[tiab] OR aEEG[tiab] OR EEG[tiab] OR electroencephalo*[tiab] OR diffusion tensor imag*[tiab] OR DTI[tiab]).

For topic #3 we used the following search terms: ("Anemia"[Mesh] OR anemi*[tiab] OR anaemi*[tiab] OR "Blood Transfusion"[Mesh] OR blood transfus*[tiab] OR erythrocyte transfus*[tiab] OR red cell transfus*[tiab] OR red blood cell transfus*[tiab]) **AND** ("Infant, Newborn"[Mesh] OR prematur*[tiab] OR preterm[tiab] OR neonat*[tiab] OR newborn*[tiab] OR infant*[tiab] OR elbw[tiab] OR vlbw[tiab]) **AND**  (“Cognition”[Mesh] OR “Intelligence”[Mesh] OR “Intelligence Tests”[Mesh] OR “Cognition Disorders”[Mesh] OR “Neuropsychological Tests”[Mesh] OR “Child Development”[Mesh] OR “Psychomotor Performance”[Mesh] OR “Neurodevelopmental Disorders”[Mesh] OR cognition[tiab] OR cognitive[tiab] OR ((neurodevelopment*[tiab] OR neurologic*[tiab] OR development*[tiab]) AND (outcome*[tiab] OR delay*[tiab] OR chang*[tiab] OR disabil*[tiab] OR functioning[tiab] OR assess*[tiab] OR test[tiab] OR tests[tiab] OR testing[tiab])) OR (neurologic*[tiab] AND development*[tiab]) OR intelligen*[tiab] OR IQ[tiab] OR neurocognit*[tiab] OR bayley[tiab]) **AND** (“Epidemiologic Studies”[Mesh] OR cohort*[tiab] OR prospective[tiab] OR longitudinal*[tiab] OR longterm[tiab] OR long-term[tiab] OR follow-up[tiab] OR followup[tiab] OR case-control[tiab]) **AND** (“Child”[Mesh] OR “Infant”[Mesh] OR “Adolescent”[Mesh] OR child*[tiab] OR infant*[tiab] OR adolescen*[tiab] OR pediatr*[tiab] OR paediatr*[tiab] OR teen*[tiab] OR youth[tiab] OR kids[tiab]) NOT (“Case Reports”[Publication Type]).

*Supplemental Tables*

**Supplemental Table 1.** Newcastle-Ottawa Quality Assessment Scale for studies on cerebral tissue oxygenation, measured by NIRS

| Study (First Author, Journal, Year of Publication) | Selection | Comparability | Exposure (Case-Control Study) or Outcome (Cohort Study) | Total Score (Max 9) |
| --- | --- | --- | --- | --- |
| Goldstein et al, *Transfusion*, 2020 | 3 | 1 | 3 | 7 |
| Whitehead et al, *Early Hum Dev*, 2019 | 2 | 1 | 3 | 6 |
| Jani et al, *Transfusion*, 2019 | 3 | 2 | 3 | 7 |
| Aktas et al, *J Paediatr Child Health*, 2019 | 3 | 1 | 3 | 7 |
| Jain et al, *Pediatr Res*, 2019 | 3 | 2 | 3 | 8 |
| Saito-Benz et al, *Acta Paediatr*, 2019 | 3 | 0 | 3 | 6 |
| Kalteren et al, *Am J Perinatol*, 2018 | 2 | 2 | 2 | 6 |
| Mintzer et al, *Am J Perinatol*, 2018 | 2 | 1 | 3 | 6 |
| Whitehead et al, *J Perinatol*, 2018 | 2 | 1 | 3 | 6 |
| Li et al, *Int J Clin Exp Med*, 2017 | 3 | 0 | 3 | 6 |
| El-Dib et al, *Am J Perinatol*, 2016 | 3 | 1 | 2 | 6 |
| Banerjee et al, *Early Hum Dev*, 2016 | 4 | 1 | 2 | 7 |
| Andersen et al, *Arch Dis Child Fetal Neonatal Ed*, 2015 | 4 | 1 | 3 | 8 |
| Mintzer et al, *J Neonatal Perinatal Med*, 2014 | 2 | 1 | 3 | 6 |
| Sandal et al, *Transfusion*, 2014 | 2 | 1 | 3 | 6 |
| Koyano et al, *Transfusion*, 2013 | 3 | 1 | 2 | 6 |
| Seidel et al, *J Perinatol*, 2013 | 3 | 1 | 3 | 7 |
| Bailey et al, *Am J Perinatol*, 2010 | 3 | 2 | 2 | 7 |
| Dani et al, *Transfusion*, 2010 | 3 | 1 | 2 | 6 |
| van Hoften et al, *Arch Dis Child Fetal Neonatal Ed*, 2010 | 4 | 1 | 3 | 8 |
| Dani et al, *Acta Paediatr*, 2002 | 3 | 1 | 3 | 7 |
| Wardle et al, *J Cereb Blood Flow Metab*, 2000 | 2 | 1 | 3 | 6 |

**Supplemental Table 2A.** Newcastle-Ottawa Quality Assessment Scale for studies on brain injury and development

| Study (First Author, Journal, Year of Publication) | Selection | Comparability | Exposure (Case-Control Study) or Outcome (Cohort Study) | Total Score (Max 9) |
| --- | --- | --- | --- | --- |
| Fontana et al, *Blood Transfus*, 2020 | 3 | 0 | 3 | 6 |
| Benavides et al, *J Matern Fetal Neonatal Med*, 2019 | 2 | 1 | 3 | 6 |
| Morris et al, *J Perinatol*, 2018 | 3 | 1 | 3 | 7 |
| McCoy et al, *Dev Neuropsychol*, 2014 | 4 | 1 | 3 | 8 |
| Nopoulos et al, *Arch Pediatr Adolesc Med*, 2011 | 3 | 1 | 3 | 7 |

**Supplemental Table 2B.** Van Tulder Scale for studies on brain injury and development

| Study (First Author, Journal, Year of Publication) | A | B | C | D | E | F | G | H | I | J | K | Total Score (Max 11) |
| --- | --- | --- | --- | --- | --- | --- | --- | --- | --- | --- | --- | --- |
| Kirpalani et al, *N Engl J Med*, 2020 | 1 | 1 | 1 | 0 | 0 | 1 | 1 | 1 | 1 | 1 | 1 | 9 |
| Franz et al, *JAMA*, 2020 | 1 | 1 | 1 | 0 | 0 | 1 | 1 | 1 | 1 | 1 | 1 | 9 |
| Chen et al, *Pediatr Neonatol*, 2009 | 1 | 1 | 1 | 0 | 0 | 1 | 1 | 1 | 1 | 1 | 0 | 8 |
| Kirpalani et al, *J Pediatr*, 2006 | 1 | 1 | 1 | 0 | 0 | 1 | 1 | 1 | 1 | 1 | 0 | 8 |
| Bell et al, *Pediatrics*, 2005 | 1 | 1 | 1 | 0 | 0 | 1 | 1 | 1 | 1 | 1 | 0 | 8 |

**Supplemental Table 3A.** Newcastle-Ottawa Quality Assessment Scale for studies on neurodevelopmental outcome

| Study (First Author, Journal, Year of Publication) | Selection | Comparability | Exposure (Case-Control Study) or Outcome (Cohort Study) | Total Score (Max 9) |
| --- | --- | --- | --- | --- |
| Fontana et al, *Blood Transfus*, 2020 | 3 | 1 | 3 | 7 |
| Wang et al, *Pediatr Neonatol*, 2017 | 3 | 1 | 3 | 7 |
| Velikos et al, *Res Dev Disabil*, 2015 | 3 | 2 | 2 | 7 |
| McCoy et al, *Dev Neuropsychol*, 2014 | 4 | 1 | 3 | 8 |
| von Lindern et al, *BMC Pediatrics*, 2011 | 3 | 1 | 3 | 7 |
| Gabrielson et al, *Acta Paediatr*, 2002 | 3 | 1 | 3 | 7 |

**Supplemental Table 3B.** Van Tulder Scale for studies on neurodevelopmental outcome

| Study (First Author, Journal, Year of Publication) | A | B | C | D | E | F | G | H | I | J | K | Total Score (Max 11) |
| --- | --- | --- | --- | --- | --- | --- | --- | --- | --- | --- | --- | --- |
| Kirpalani et al, *N Engl J Med*, 2020 | 1 | 1 | 1 | 0 | 0 | 1 | 1 | 1 | 1 | 1 | 1 | 9 |
| Franz et al, *JAMA*, 2020 | 1 | 1 | 1 | 0 | 0 | 1 | 1 | 1 | 1 | 1 | 1 | 9 |
| McCoy et al, *Child Neuropsychol*, 2011 | 1 | 1 | 1 | 0 | 0 | 1 | 1 | 1 | 1 | 1 | 0 | 8 |
| Whyte et al, *Pediatrics*, 2009 | 1 | 1 | 1 | 0 | 0 | 1 | 1 | 1 | 1 | 1 | 1 | 9 |
